# The mechanisms of feature inheritance as predicted by a systems-level model of visual attention and decision making

**DOI:** 10.2478/v10053-008-0019-y

**Published:** 2008-07-15

**Authors:** Fred H. Hamker

**Affiliations:** Department of Psychology, Westf.-Wilhelms-Universität Münster, Germany

**Keywords:** feature inheritance, attention, decision making, feedback, VSTM, computational model

## Abstract

Feature inheritance provides evidence that properties of an invisible target stimulus can be attached to a following mask. We apply a systemslevel model of attention and decision making to explore the influence of memory and feedback connections in feature inheritance. We find that the presence of feedback loops alone is sufficient to account for feature inheritance. Although our simulations do not cover all experimental variations and focus only on the general principle, our result appears of specific interest since the model was designed for a completely different purpose than to explain feature inheritance. We suggest that feedback is an important property in visual perception and provide a description of its mechanism and its role in perception.

## Introduction

The perception of a briefly flashed target stimulus followed by a mask can be strongly impaired or, depending on the mask and the stimulus-onset asynchrony, the stimulus can be easily detectable. Theories of visual masking explain the impaired perception typically by an erosion of the target information, be it by temporal fusion, interruption or suppression through competition. In feature inheritance, however, the mask inherits a property of the target stimulus (e.g. Herzog & Koch, 2001). For example, a vernier, a tilted line, or a bar in apparent motion are presented for a short time and followed immediately by a grating comprising a small number of straight elements. The grating is perceived as offset, tilted, or moving. The perceived distortion (e.g. tilt) is much smaller than the actual property of the target. The target stimulus itself remains largely invisible. This effect cannot be easily explained by a simple temporal fusion since the property of the mask is only slightly distorted and the effect lasts for mask presentation times of about 300 ms. Moreover, when target and mask are very different in orientation, both appear visible (shine through). Thus, feature inheritance demonstrates that stimulus properties can act upon the properties of a following stimulus.

The mechanism responsible for feature inheritance is still unclear, but some recent work addressed its neural correlate. Zhaoping (2003) explains feature inheritance by lateral figure-ground binding in V1 and shows that a vernier followed by a grating consisting of a few elements results in only one or two saliency peaks at the border of the grating, whereas a grating with several elements results also in a saliency peak at the center, suggesting no feature inheritance but shine through. However, the actual decoding of this saliency information into a percept or a decision has not been modeled and it remains open in how far V1 saliency is responsible for the perception of an offset or tilt. We have recently developed a computational model to explain most of the temporal phenomenology of feature inheritance (Ma, Hamker, & Koch, 2006). We varied the duration of target and mask presentation and tuned the parameters of the model to be consistent with observations. According to the model, a subsystem creates an inert hypothesis about the stimulus which is then tested against the later input. Cells further downstream, related to object perception, only fire when the hypothesis is confirmed. We will call this a strong hypothesis testing model. Although the model can account for several observations, the hypothesis-testing subsystem was specifically designed to explain feature inheritance. While this approach is typical for most computational models, fundamental insights can only be achieved if a model generalizes to other phenomena. Thus, we here apply a model of visual attention to the paradigm of feature inheritance to gain further insight into general mechanisms of visual perception. This model contains a mechanism of weak hypothesis testing by means of feedback, which implements feature-based attention and goal-directed search and resolves ambiguities (Hamker, 2005a; Hamker, 2005b; Hamker, 2006). Weak hypothesis testing refers to the rule according to which feedback is not necessary for brain areas to process the stimulus-driven feedforward signal. Feedback only modulates processing.

Object substitution theory proposes that masking is a consequence of ongoing recurrent interactions between different levels of the cortical hierarchy (Di Lollo, Enns, & Rensink, 2000; Enns, 2002). The first stimulus is initially processed in a feedforward sweep. This sweep activates neurons at high levels which project back to earlier levels. With respect to feature inheritance, the features of a target can be incorporated into the activation pattern of a following mask if both are similar (Enns, 2002). At this level of abstraction, our model is very similar, if not identical, to object substitution theory. However, one key idea of the object substitution theory is that perception requires a confirmation of the perceptual hypothesis by comparing the hypothesis at the higher level with the ongoing activity at the lower level (Enns, 2002; Di Lollo et al., 2000). The exact mechanism of this comparison is critical, and requires a clear definition. Although, feedback has been emphasized in several models of visual perception, its exact mechanism significantly differs across these models. In the computational model of object substitution (CMOS) the input into the higher area is defined as the sum of feedback and feedforward (Di Lollo et al., 2000). A summation predicts the activation of cells at an early level by feedback from higher levels and thus, both, the actual signal and the top-down hypothesis are simultaneously activated at an early level.

Several approaches treat vision as a generative process (Mumford, 1992; Olshausen & Field, 1997; Rao, 1999). According to this paradigm, feedback represents the predicted image and the feedforward signal the residual image which is obtained by subtracting the predicted image from the input image. A good match between the internal hypothesis and the actual input results in a weak feedforward signal and a mismatch in a strong signal. Thus, feedback primarily serves to “explain away” the evidence by suppressing the activity. This approach has been primarily used for the learning of receptive fields and object recognition. Its relevance for masking or feature inheritance has not been explored so far.

Our approach, which shows some similarity to adaptive resonance (Grossberg, 1980), interactive activation models (McClelland & Rumelhart, 1981), Bayesian belief propagation and particle filtering (Lee & Mumford, 2003), predicts an enhancement if both signals are consistent with each other by increasing the gain of the feedforward signal. If both signals are not consistent no enhancement occurs, i.e., no gain change takes place. Perception in our model can be actively guided by an internal hypothesis, but a match between the visual observation and the internal hypothesis is not required for the activation of visual areas (weak hypothesis testing approach). Thus, a purely sensory-driven activation (with and without feedback) is sufficient to activate all model areas. Due to competitive interactions irrelevant information is inhibited (Hamker, 2004), similar as in the Biased Competition framework (Desimone & Duncan, 1995). We have termed this interaction of the top-down or feedback with the feedforward signal as population-based inference (Hamker, 2005a; Hamker, 2005b), since it implements an inference operation but differs in several aspects from a true Bayesian approach. In the following we will briefly introduce the model of attention and its mechanism of feedback. We then apply different versions of the model to simulate a typical feature inheritance experiment and derive conclusions about the role of feedback and memory in visual perception. The fact that human subjects can under some conditions report a masked, briefly flashed stimulus has lead to two alternative interpretations (Smith, Ratcliff, & Wolfgang, 2004). In the first one, stimulus properties get encoded in visual short-term memory (VSTM), and its content represents the input for the decision process. In the second one, the decaying iconic trace provides the input for decision making. We will also discuss a third alternative. Here, memory provides a top-down signal which modifies the properties of visual areas. The decision however, is still based on the content of the iconic trace. We call this approach active hypothesis testing.

We are specifically interested in the question if memory-based, active hypothesis testing is required for feature inheritance to occur, or if passive hypothesis testing by feedback, is sufficient. Thus, we have tested five different models, two where perception is only sensory-driven, and three where perception is hypothesis-driven. We obtain an internal hypothesis by memorizing a representation of the stimulus at different times. From the two models of sensory-driven perception, one can be categorized as passive hypothesis testing, since it contains feedback but no external top-down signal. In the other one, we removed feedback.

## METHODS

### Systems-level model of attention

 Our model of attention is an extension of an earlier model (Hamker, 2003; Hamker, 2004; Hamker, 2005a), which has been strongly constrained by several electrophysiological observations and anatomy. The present version operates with real input images. It has been applied on tasks such as object detection in natural scenes, change detection, visual search, and feature-based attention (Hamker, 2005b; Hamker, 2005c; Hamker, 2006). Since it has been extensively described in Hamker (2005b) we here give only a brief overview with emphasis on the relevant aspects for feature inheritance. 

**Figure 1.**
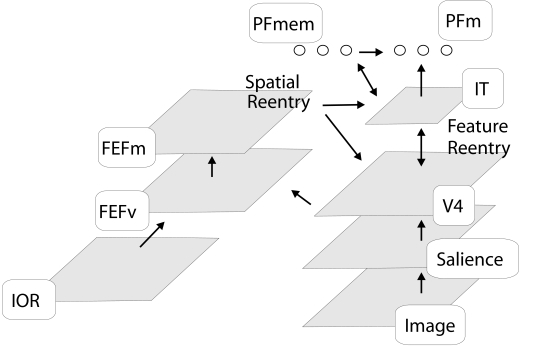
Model for visual attention. First, information about the content and its low level stimulus-driven salience is extracted. (Stimulus-driven saliency, however, will not be crucial for the results obtained here.) This information is sent further downstream to V4 and to IT cells which are broadly tuned to location. A target template is encoded in PF memory (PFmem) cells. Feedback from PFmem to IT increases the strength of all features in IT matching the template. Feedback from IT to V4 sends the information about the target downwards to cells with a higher spatial tuning. FEF visuomovement (FEFv) cells combine the feature information across all dimensions and indicate salient or relevant locations in the scene. The FEF movement (FEFm) cells compete for the target location of the next eye movement. The activity of the FEF movement cells is also sent to V4 and IT for gain modulation. However, in all simulations we set the model to fixate, which results in a suppression of the FEF movement activity. The IOR map is not used for the experiments simulated here.

The model consists of visual areas V4, inferotemporal (IT) cortex, prefrontal areas that contain the frontal eye field (FEF) for saccade planning and more ventrolateral parts for implementing functions of working memory (Fig. 1). If we present a visual scene to the model, features such as color, intensity and orientation are computed from the image. We will here consider only the orientation channel.

Search in this model can be goal directed since IT receives feature-specific feedback from the prefrontal memory (PFmem) cells. Feedback from the IT in turn increases the gain of the cells in V4. Because of the growing receptive filed size from V4 to IT many V4 cells receive feedback from a single IT cell.

The planning of an eye movement is implemented as follows. The FEF visuomovement (FEFv) neurons receive afferents from V4 and IT. The input activity at each location is summed across all dimensions (e.g. color, orientation). The firing rate of FEF visuomovement cells represents the saliency and task relevance of a location. The FEF movement cells compete for the selection of the strongest location. If a FEF movement cell exceeds a threshold, an eye movement is indicated. In the simulation of the feature inheritance effect the model is set into fixation by a continuous inhibition of the movement cells.

### Population-based inference

We have developed a population-based inference approach to implement the top-down guidance of vision by internal expectations. Decision making involves uncertainty arising from noise in sensation and the ill-posed nature of perception. Thus, alternative interpretations should be represented until a decision is found. Such constraints can be well handled by a population code. It offers a dual coding principle. A feature is represented by the location of a cell i within the population, and the conspicuity of this feature is represented by the firing rate ri. The change of the firing rate is described by the following differential equation:

(1) $$\tau \frac{d}{d\tau }r_{i}=I_{i}^{↑}+I_{i}^{↔}+I_{i}^{↓}-(r_{i}+a)I^{inh}$$

The conspicuity represents the accumulated evidence and reflects stimulus-driven saliency as well as task relevance. The input is a result of bottom-up input *I^↑^* a modulated by lateral *I^↔^* and top-down influence. *I^inh^* represents a weighted sum of all the activity in the population. Thus, *(r_i_ + a)I^inh^* leads to a competition among the cells, such that a gain enhancement for some cells results in a mild suppression for other cells. The suppression depends on the activity *r_i_* and on the parameter *a* (e.g.,*a* = 0.1).

*I^↓^* defines how the integrated stimulus representation is continuously updated using prior knowledge in form of generated expectations. The idea is that all mechanisms act directly on the processed variables and modify their conspicuity. Thus, attending a certain feature or a location in space enhances the probability of a feature being detected.

The integrated representation of the bottom-up observation *I^↑^_i_* and the top-down expectation ${\tilde{r}}_{i}$ is obtained by a gain modulation of the bottom-up observation. If the observation is similar to the expectation the conspicuity (firing rate) of the integrated representation is increased by

(2) $$I_{i}^{↓}=I_{i}^{↑}\cdot {\left[A-\underset{i}{\max(r_{i})}\right]}^{+}w{\tilde{r}}_{i}$$

**Figure 2.**
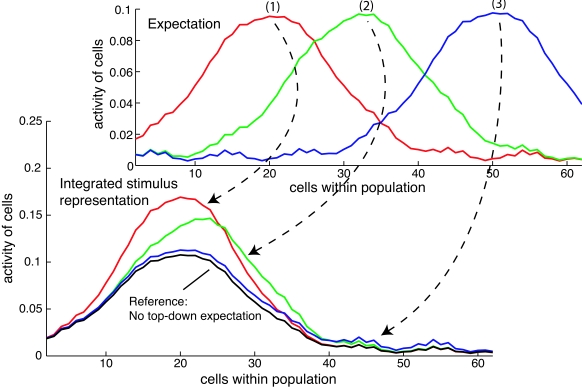
Population-based inference using three different expectations. The x-axis represents the feature space such as orientation, and the y-axis represents the firing rate of the cells. (1) When the expectation is equal to the observation, the conspicuity of the integrated stimulus representation is enhanced as compared to the unmodulated reference. (2) A partial overlap of expectation and observation results in the distortion of the population response into the direction of the expectation. However, the distorted response still primarily encodes the information from the observation. (3) When the expectation is much different, the integrated stimulus representation is largely unchanged.

As long as the maximal activity within the population is lower than a threshold (e.g. *A*=1), the feedback signal ${\tilde{r}}_{i}$ effectively increases the gain. On the population level, however, the local gain mechanism can result in the distortion of the population response and thus in a misperception. Figure 2 illus trates three different cases obtained by simulations using additional noise. When the expectation ideally matches the observation (case 1), the integrated stimulus representation reflects primarily an increase in conspicuity. When the expectation only partially matches the observation (case 2), the population response is distorted and reflects a compromise between the observation and the expectation. This is different from a Bayesian inference approach, where the estimated response can also primarily follow the expectation, if the probability density distribution for the expectation is very narrow and the one for the observation is very broadly tuned. When the expectation is much different from the observation (case 3), the top-down signal has almost no direct influence on the population response. Thus, feedback in population-based inference is a weak form of hypothesis testing. In the simulation results shown in Fig. 2 the top-down expectation is independent from the bottom-up input and not connected within a loop as it is in the model. When both are connected with each other and no additional permanent top-down input exists, the integrated population response will finally reflect the observation if we wait sufficiently long enough.

We have recently shown that our population based inference approach is general enough to explain also spatial effects such as the shift and shrinkage of receptive fields in area V4 prior to saccade (Hamker & Zirnsak, 2006).

### Simulation of the feature-inheritance experiment

**Figure 3.**
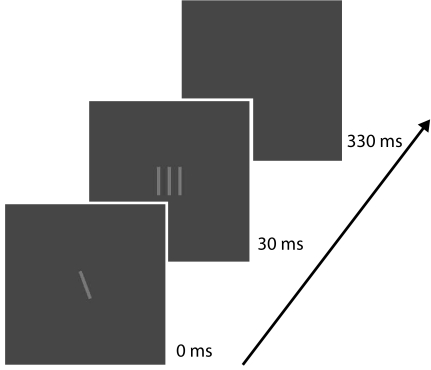
We used images of 300x300 pixel in size, where each bar is 26x6 pixel in size. A target stimulus was presented for 30 ms followed by a mask shown for 300 ms. After the mask, a blank image was presented to the model. The relative orientation of the target to the mask was varied (0°, 5°, 10°, 15°, 20°, 25°, 30°, 40°, 45°, 50°, 55°, 60°) to investigate the dependency of feature inheritance on the similarity in the feature space.

 We used a similar experimental procedure as Herzog and Koch (2001) . The original sequence of images presented to the model is shown in Figure 3. The target is visible for 30 ms (simulation time) followed by a grating for another 300 ms. After 330 ms the input switches to a gray image, allowing us to simulate the decay of activity as well. It has been earlier suggested that some aspects of masking depend on principles related to the Gestalt (Herzog, Ernst, Etzold, & Eurich, 2003). Since our model does not contain comprehensive algorithms for grouping, we omit simulations with different numbers of bars in the mask and focus primarily on the orientation similarity of the target and the mask. Thus, we varied the relative orientation of the target to the mask using 12 different target orientations (0°, 5°, 10°, 15°, 20°, 25°, 30°, 40°, 45°, 50°, 55°, 60°).

The model has been set to avoid overt and covert shifts of spatial attention. The only mechanisms active are all feedforward connections, feature-based feedback from PFmem to IT and from IT to V4. The PFmem cells are typically used for goal-directed visual search. They hold a target template which changes the gain of IT cells throughout a trial. In the simulation of sensory-driven perception the PFmem cells can be activated but the pattern is not memorized and the neural activation changes with the input. Since perception might activate an internal hypothesis used to guide the visual system (Lleras, Rensink, & Enns, 2005; Hamker, 2005a), we simulate three conditions where the IT activation is memorized in PFmem cells for an ongoing active hypothesis testing. We used a memorization at 100-120 ms, 140-160 ms and 180-200 ms. After this memorization period the content of the PFmem cells is not subject to change and continuously influences IT activity.

### Decision making

 Our model allows us to simulate the temporal course of activity in different brain areas. In order to close the gap between a continuous time varying signal and a finite decision of a human subject we will use a simple neural decision model, which reads out the population response in the orientation channel and determines if the mask is perceived as tilted or not. Models of decision making that accumulate the evidence over time have a long tradition in mathematical psychology leading to several models. For an overview see Smith and Ratcliff (2004) as well as Usher and McClelland (2001) and for a comparison of models refer to Ratcliff and Smith (2004) . Despite many differences the general idea is very similar. All models accumulate the evidence from a time-varying input signal and stop when a criterion is reached such as the crossing of a threshold. In most decision making simulations the input of the model is not a true time-varying signal but obtained from probability distributions. Our model is similar to the leaky, competing accumulator model of Usher and McClelland (2001) . However, Usher and McClelland (2001) simplify the input of their model to ensure a convergence by setting the sum of all inputs equal to one. The differences of our model to theirs are primarily required by the constraint that we directly use the neural activity in model IT to determine the evidence for either choice. 

**Figure 4.**
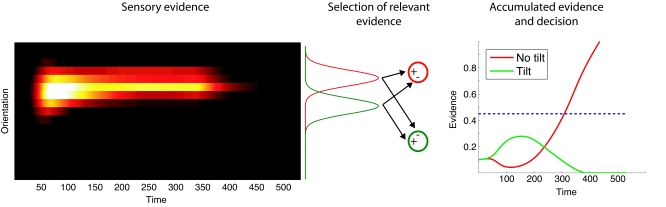
Accumulation of sensory evidence and decision. The neural activity in the orientation channel provides the sensory evidence about the presented visual scene. We weighted this activity with respect to the preferred orientation of the cells using a Gaussian function to determine the specific evidence for the decision “tilt” and “no tilt”. The present selected, sensory evidence for one hypothesis is subtracted from the selected, sensory evidence for the other hypothesis. The accumulated evidence in the competing accumulator model is compared to a decision threshold (dashed line) to obtain the final decision.

Subjects probably learn what information is relevant in a particular experimental situation. In our model, we select the relevant information by weighting the activity, distributed across the feature space, with a Gaussian (Fig. 4). In order to keep this selection process simple, we hold the parameters fixed for all simulations. The parameters have been determined to allow a robust decision between *tilt* and *no tilt*. Thus, the weight of the projection from a cell *i* encoding the orientation of the stimulus to a cell *j* involved in the decision is

(3) $$w_{ij}=e^{\frac{\|u_{i}-{c_{j}\|}^{2}}{2\sigma^{2}}}$$

where ui is the preferred orientation of the cell *i* and *c_j_* is the center of the Gaussian relative to the orientation of the mask (*c_tilt_* = 6°; *c_no tilt_* = -3°; σ = 10° |*tilt* = 1, *no tilt* = 2). The input for each choice is then

(4) $$\begin{array}{l} I_{1}=\sum_{i}w_{i1}r_{i}^{IT}-\sum_{i}w_{i2}r_{i}^{IT}\\ I_{2}=\sum_{i}w_{i2}r_{i}^{IT}-\sum_{i}w_{i1}r_{i}^{IT} \end{array}$$

following the common approach that the evidence for one choice reduces the evidence of the other choice (Mazurek, Roitman, Ditterich, & Shadlen, 2003). The accumulated evidence is computed within a laterally connected set of two neurons *r*_1_ and *r*_2_:

(5) $$\tau \frac{d}{d\tau }r_{1}(t)=I_{1}(k+w^{+}\cdot r_{1}(t))+a\cdot w^{+}\cdot r_{1}(t)-w^{-}\cdot r_{1}(t)r_{2}(t)$$$$\tau \frac{d}{d\tau }r_{2}(t)=I_{2}(k+w^{+}\cdot r_{2}(t))+a\cdot w^{+}\cdot r_{2}(t)-w^{-}\cdot r_{2}(t)r_{1}(t)$$

with *k* = 1.5: *w*^+^ = 4; *w*^–^ = 0.1; *a* = 0.04; τ = 50 and an initial value of *r*_1_(0) = *r*_2_(0) = 0.1. The cell that first crosses a threshold (γ = 0.45) determines the decision and the time of the crossing represents the internal reaction time (excluding the time for the overt response). Our model converges in all cases to a final decision, even when the evidence during a period of time is very similar for each choice. Since we primarily want to use this model as a tool to evaluate the encoded information in the model of attention, the simulations of the decision process are performed without additional noise.

## RESULTS

**Figure 5.**
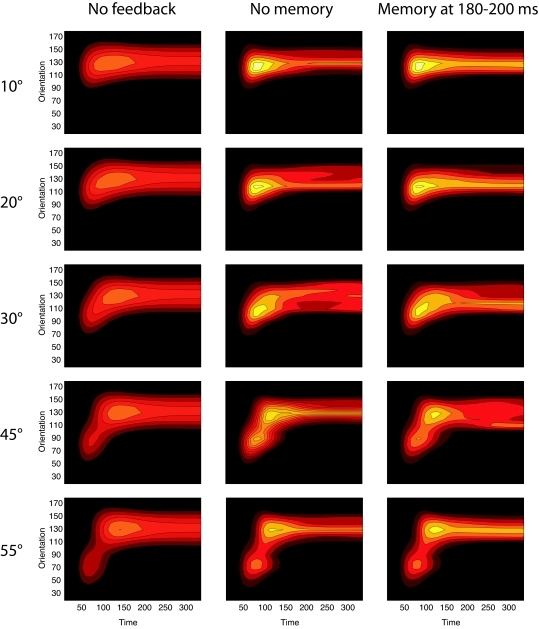
Population activity in IT from target onset to mask offset in three different model conditions, sensory-driven perception without feedback, sensory driven perception with feedback and hypothesis-driven feedback with the memorizing a target template at 180-200 ms after target onset. The numbers on the left indicate the orientation offset of the target stimulus with respect to the mask.

**Figure 6.**
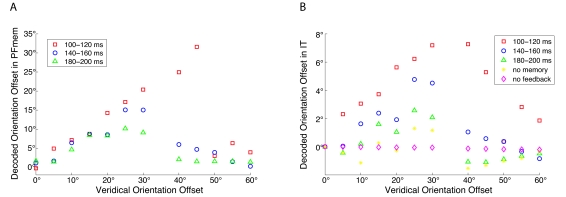
Encoded orientation information in the population activity at 300 ms after target onset with respect to the veridical orientation. The decoding of the encoded orientation in the population response has been done with a simple population vector method (Dayan and Abbott, 2001). (**A**) Decoded orientation relative to the mask in the PFmem cells. The memorization of the IT activity at different times reflects the sustained influence of the briefly presented target on the population response. The sustained influence is orientation dependent. If the orientation of target and mask differ strongly the information from the target is not memorized. Only when the memorization of the IT activity occurs at 100-120 ms, a target stimulus of an orientation offset of 40° or larger largely distorts the population. For orientation differences up to 30° some information of the target is still encoded by the population. (**B**) The population response in IT receives a small but sustained distortion, if a template has been memorized and used for top-down guidance. In the models with no memory or without feedback the information from the target stimulus has faded away at 300 ms after target onset. Note, the y-axis in panels A and B scales differently.

We simulated five different models, (1) sensory-driven without feedback, (2) sensory-driven with feedback (passive hypothesis testing), and three versions of active hypothesis testing (3) hypothesis-driven with memory encoding between 100-120 ms, (4) hypothesis-driven with memory encoding between 140-160 ms, and (5) hypothesis-driven with memory encoding between 180-200 ms. For each model we ran 12 trials with a varying orientation offset between target and mask (0°, 5°, 10°, 15°, 20°, 25°, 30°, 40°, 45°, 50°, 55°, 60°). In the simulation of the model without feedback the cells in IT fire less vigorously (Fig. 5). However, more important appears the general trend that the peak activity is shifted to the orientation of the target when we compare the model without feedback to other models. At an orientation offset of about 45° or more, a second peak in the population response emerges. We did not test if our decision model can detect this peak since the alternative choice is poorly defined, but it appears that in this case the target is either successfully masked or shines through the mask. Without feedback, the information of the target is erased at 100-150 ms depending on the orientation offset, whereas with feedback the information erases between 150-200 ms after target onset. Thus, the memorization of the neural response at different times leads to less target information in memory with increasing time (Fig. 6A). Moreover, for all three models of hypothesis-driven perception, large orientation offsets lead to little or no influence of the target information on the population encoded in memory since only the strongest population enters memory. According to the first approach to the perception of masked visual stimuli, the memory content represents the input of the decision (Smith et al., 2004). Thus, this model predicts the perception of relatively strong tilts (Fig. 6A). In many cases, the perceived tilt is about half of the veridical tilt, which is not consistent with the typical observation (Herzog & Koch, 2001).

If we now consider the third approach to the perception of masked visual stimuli where memory modifies visual areas we observe for all three models that the IT activity is permanently distorted towards the target orientation (Fig. 6B). The strength of the distortion depends on the content in memory and thus on the time of memory encoding. Furthermore, the tilt is only relatively small. Thus, the late response in hypothesis-driven perception is dominated by the mask but slightly distorted towards the target, if target and mask orientation are sufficiently similar to each other.

**Figure 7.**
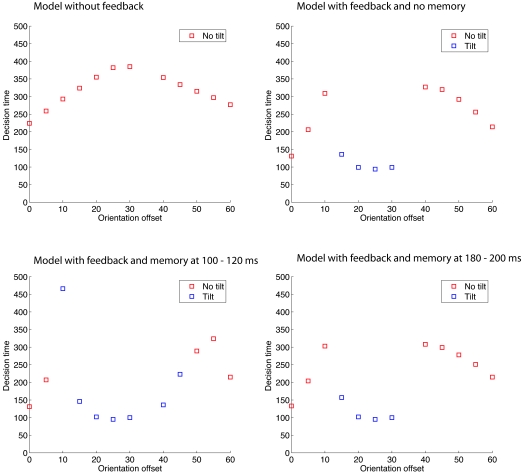
Population activity in IT from target onset to mask offset in three different model conditions, sensory-driven perception without feedback, sensory driven perception with feedback and hypothesis-driven feedback with the memorizing a target template at 180-200 ms after target onset. The numbers on the left indicate the orientation offset of the target stimulus with respect to the mask.

The present results suggest that feature inheritance requires hypothesis-driven perception (active hypothesis testing) where memory permanently distorts the response in IT. The effect also occurs on the level of V4 but to a lesser degree. However, we did not look at the properties of the second approach to the perception of masked visual stimuli, in which the decaying iconic trace feeds the perceptual decision. A sustained distortion of the population response might not be necessary, if we consider that a perceptual choice is made by the accumulation of evidence. Thus, we fed the evidence for a tilted and non-tilted neural response into a model of decision making and determined the response and time of decision (Fig. 7). The perception of a tilt is an indicator for feature inheritance. No tilt either reflects complete masking or shine through. In the sensory-driven perception without feedback no tilt of the mask has been detected. In the sensory-driven perception with feedback, however, the model responds the perception of a tilt for an orientation difference of 15°-30°. The model of hypothesis-driven perception with memory encoding between 180-200 ms and the one with memory encoding between 140-160 ms (not shown) respond almost equal in decision and response time than the model of sensory-driven perception with feedback (passive hypothesis testing). If the memory encoding occurs earlier in time (100-120 ms), the model predicts the perception of a tilt from an orientation offset of 10°-45°. The difference between the two models of sensory-driven perception has not been obtained by a clever adjustment of the decision threshold. For all orientations, in the model without feedback the accumulated evidence for a tilted grating was never close to the threshold. Thus, feedback appears necessary and sufficient for feature inheritance to occur, of course, depending on the timing and similarity of target and mask.

Perceptual decision based on the accumulated sensory evidence in four different models. In the model without feedback the model predicts no tilt in all conditions. The models with feedback, either with or without memory predict the perception of a tilt, depending on the orientation offset. The decision time for the perception of a tilt is in most conditions very fast.

## DISCUSSION

With regard to the role of VSTM in the perception of masked visual stimuli we do not find support for the first explanation according to which the content of VSTM provides the input of the decision, since our model VSTM predicts the perception of a strong tilt (Fig. 6A). Although this effect varies with the time of memory encoding, the encoding at 180-200 ms still predicts the perception of a relatively strong tilt. Our results are more consistent with the idea that the iconic trace provides the input for decision making, either with or without the influence of VSTM. The observation that the perception of a tilt or offset varies largely across subjects (Herzog & Koch, 2001) might depend on their decision criterion. Subjects which are trained in fast decision making, such as playing ball games might use a low threshold and thus they perceive an influence of the target. In subjects using a conservative criterion (high threshold), the mask dominates the decision and the subject does not perceive the tilt, or the target presentation times have to be longer. This view of perceptual decision making is similar to masked response priming which can also be modeled by a neural accumulation process (Vorberg et al., 2003).

Somewhat surprisingly is our observation that feedback-loops alone are sufficient to lead to feature-inheritance. Although the information of the target disappears at about 150-200 ms after target onset, feedback holds the target information sufficiently long to influence the decision with respect to the perceived orientation. We do not claim that feature inheritance necessarily occurs at the level of IT and V4. Our proposed feedback mechanism is a general mechanism of feedback and also acts from V2 to V1 and V4 to V2. Consistent with observations, the model predicts that feature inheritance only occurs within a limited range of an orientation difference between target and mask. Since we only used 20 cells to represent the orientation space and did not tune the width of the population response the exact range might be slightly different, e.g., subjects reported feature inheritance if elements are tilted by 7° (Herzog & Koch, 2001). At the level of the decision, the model of sensory-driven perception does not fundamentally differ from the model of hypothesis-driven perception. However, the model of sensory-driven perception without feedback does not provide sufficient evidence for a feature-inheritance effect. From our analysis we cannot exclude that other mechanisms than feedback can also account for feature-inheritance. The strength of our approach rather lies in its generality. Our model was designed for a completely different purpose, but nevertheless, without modification, it shows a feature-inheritance effect. We acknowledge that a comprehensive demonstration of the role of feedback in feature inheritance requires more simulations and perhaps also changes in the model, but at present, it appears important to us to identify general, universal mechanisms of perception as compared to specialized models tuned to a single experimental paradigm, such as our earlier model (Ma et al., 2006). Our model appears also consistent with the observation of a trace carried over a sequence of invisible elements (Otto, Öğmen, & Herzog, 2006). Other experiments have revealed that the locus of spatial attention influences feature inheritance (Sharikadze, Fahle, & Herzog, 2005). Offsets at the attended edge of the grating influence performance whereas offsets of non-attended elements do not show a strong influence. This is probably not easy to test with orientations, since local orientation differences typically pop-out. However, these results provide additional constraints for models of feature inheritance.

The present discussion about models of visual perception is dominated by extremes such as purely feedforward models and models that require reentrant processing already at intermediate levels of visual processing. Our model provides a compromise between these extremes. It supports the feedforward sweep hypothesis (Lammé & Roelfsema, 2000; Rousselet, Thorpe, & Fabre-Thorpe, 2004), since no attention or other top-down signals are required for a stimulus being processed. Feedback can lead to the accumulation of further evidence by enhancing a specific subset of the neuronal activity or by indirectly suppressing other activity. From the anatomical point of view feedback connections are as prominent as feedforward connections (Rockland, Saleem, & Tanaka, 1994). Furthermore, feedback can act as fast as 10 ms (Hupé, James, Girard, Lomber, Payne, & Bullier, 2001). Given that a final decision typically requires to integrate information over time, there is little room for a decision purely based on feedforward evidence. We rather suggest the following scenario: Perceptual decisions are based on the accumulation of evidence over time. If the feedforward sweep of processing provides no conflicting information, the accumulation of evidence can be very fast and only little recurrent processing takes place. Indeed our framework of population-based inference predicts that the feedback signal is less effective if the neuronal activity is already high. Conflicting evidence slows down the decision process, but reentrant processing enhances the relevant information and suppresses the irrelevant. Exhaustive reentrant processing is not a prerequisite for detection and recognition. However, reentrant processing automatically kicks in and facilitates perception. Thus, a comprehensive model of the time course of visual perception should consider the role of feedback.

Other phenomena, such as the change of temporal perception, might also depend on feedback. Our model predicts a decrease in the time for a perceptual decision, if target and mask are similar. Two aspects of our model seem to be primarily involved in this speed up. First, the reentrant connections in the visual areas and second, the integration of the relevant features for the perceptual decision. Present evidence suggests, that not the pure similarity of features, but the task relevance of the features is the cause of enhanced processing speed (Scharlau & Ansorge, 2003; Enns & Oriet, 2007; Scharlau, 2007). Thus, it appears that the integration of the relevant features, i.e. the evidence, is the crucial process involved in the increase of processing speed. In the present version of our model the definition of which features are relevant is predetermined. It would be very interesting to explore how learning could lead to an automatic selection of relevant features for a given task.

Feedback might also be crucial for the relatively long duration of iconic memory, a high-capacity form of storage, lasting for at least a few hundred milliseconds (Coltheart, 1983). Iconic memory seems to be essential for visual awareness (Koch, 2004), probably by providing the substrate for the collection of evidence. This transfer from iconic memory to visual awareness is not understood so far. It is not clear if integration alone (sensory-driven perception) is sufficient or if a form of active hypothesis testing is required, as suggested by inattentional blindness experiments (Mack & Rock, 1998). The fact that passive hypothesis testing seems to be sufficient to explain feature inheritance by our model does not exclude the possibility that at a higher level, such as the transition to awareness, active hypothesis testing is required. However, is appears unlikely that a strong form of hypothesis testing occurs early in the visual pathway.

Since our model is very simple with respect to the shape of objects the present version does not allow strong predictions in other masking paradigms. However, since classical models of backward masking (Breitmeyer, 1984; Breitmeyer & Öğmen, 2000; Öğmen, Breitmeyer, & Melvin, 2003) are based on local, lateral connections, it might be interesting to further explore the role of feedback in masking. Object substitution theory provides a first important step into this direction. However, object substitution is at present a more general framework and it requires a clear definition of many underlying computational mechanisms. Our model could lead to a partial refinement of object substitution, since we have given evidence that the mechanism of feedback can be well described as a gain increase on the feedforward signal. Anyway, more detailed neural models with feedback appear a promising tool to further study the role of feedback in masking.
